# Tuning the Elasticity of Polymersomes for Brain Tumor Targeting

**DOI:** 10.1002/advs.202102001

**Published:** 2021-08-22

**Authors:** Meng Zheng, Qiuli Du, Xin Wang, Yuan Zhou, Jia Li, Xue Xia, Yiqing Lu, Jinlong Yin, Yan Zou, Jong Bae Park, Bingyang Shi

**Affiliations:** ^1^ Henan and Macquarie University Joint Centre for Biomedical Innovation School of Life Sciences Henan University Kaifeng 475004 China; ^2^ Henan Key Laboratory of Brain Targeted Bio‐nanomedicine School of Life Sciences & School of Pharmacy Henan University Kaifeng 475004 China; ^3^ School of Engineering Faculty of Science and Engineering Macquarie University Sydney NSW 2109 Australia; ^4^ Department of Biomedical Sciences Faculty of Medicine & Health Sciences Macquarie University Sydney NSW 2109 Australia; ^5^ Department of Cancer Biomedical Science Graduate School of Cancer Science and Policy National Cancer Center Goyang 10408 South Korea

**Keywords:** active targeting, brain tumor, elasticity, polymersomes

## Abstract

Nanoformulations show great potential for delivering drugs to treat brain tumors. However, how the mechanical properties of nanoformulations affect their ultimate brain destination is still unknown. Here, a library of membrane‐crosslinked polymersomes with different elasticity are synthesized to investigate their ability to effectively target brain tumors. Crosslinked polymersomes with identical particle size, zeta potential and shape are assessed, but their elasticity is varied depending on the rigidity of incorporated crosslinkers. Benzyl and oxyethylene containing crosslinkers demonstrate higher and lower Young's modulus, respectively. Interestingly, stiff polymersomes exert superior brain tumor cell uptake, excellent in vitro blood brain barrier (BBB) and tumor penetration but relatively shorter blood circulation time than their soft counterparts. These results together affect the in vivo performance for which rigid polymersomes exerting higher brain tumor accumulation in an orthotopic glioblastoma (GBM) tumor model. The results demonstrate the crucial role of nanoformulation elasticity for brain‐tumor targeting and will be useful for the design of future brain targeting drug delivery systems for the treatment of brain disease.

## Graphical abstract

Elastic tunable polymersomes, prepared by self‐assembly of poly(ethylene glycol)‐*block*‐poly(pentafluorophenyl methacrylate) (PEG‐*b*‐P(PFPMA)) block copolymers and crosslinked with different diamine crosslinkers. These crosslinked polymersomes with identical particle size, zeta potential, and morphology but varied with elasticity, shows different blood circulation, blood brain barrier penetration, cell uptake, tumor penetration, and tumor accumulation performance toward brain tumor.

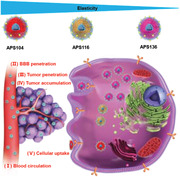

## 1 Introduction

Brain tumors are the deadliest form of cancer.^[^ ^1^ ^]^ Despite advances in drugs development and clinical technology, the prognosis of glioblastoma (GBM, the most aggressive type of brain tumor) patients remains poor, with a median survival of merely 12–15 months.^[^ ^2^ ^]^ Administration of chemical drugs, proteins, nucleic acids are convenient and promising therapeutic approaches for brain tumor therapy. However, the effectiveness of these drugs for brain disease treatment is seriously hindered by poor blood brain barrier (BBB) penetrance and lack of specific tumor targeting, short biological half‐life, as well as ineffective cellular take up etc.^[^ ^3^ ^]^ Moreover, lack of specificity, of these agents can trigger serious toxicity in normal tissues or cells.^[^ ^4^ ^]^ Nanoformulation‐based delivery solutions present a promising opportunity to greatly improve drug therapy for brain tumors.^[^ ^5^ ^]^ Indeed, intensive investigations have demonstrated that functionalized nanoformulations can be employed as smart delivery systems to enhance therapeutic outcomes and minimize sideeffects.^[^ ^6^ ^]^

Despite the promising potential of nanoformulations for drug delivery and their corresponding intensive preclinical studies, the ratio of successful clinical translation of nanomedicines is still low, reflecting limited therapeutic efficacy. In this regard, chemical strategies such as ligand modification to increase the specificity of nanomedicine toward tumor tissues has been widely utilized.^[^ ^7^ ^]^ Optimization of physical parameters was also investigated to improve drug delivery of nanoformulations. These studies showed that nanoformulation physical properties such as size,^[^ ^8^ ^]^ shape,^[^ ^9^ ^]^ surface chemistry,^[^ ^10^ ^]^ and elasticity^[^ ^11^ ^]^ play critical roles in regulating nano‐bio interactions including cell uptake, blood circulation, tumor penetration, and accumulation. Understanding how physical properties affect underlying biomechanisms should shed light on the design of drug delivery vectors with higher efficiency. Note that the effects of size, shape, and surface charge of nanoformulations on biological performance has been extensively investigated, whereas the role of nanoformulation elasticity in drug delivery is still under‐explored.

The elasticity is the ability of an object resist deformation caused by stress and to return to its original state when the stress is removed.^[^ ^12^ ^]^ The factors that may potentially impact the elasticity of nanoformulations include the intrinsic property of materials,^[^ ^13^ ^]^ crosslinking density,^[^ ^14^ ^]^ thickness,^[^ ^15^ ^]^ polymer volume fraction^[^ ^11e^ ^]^ and the encapsulated components^[^ ^11^, ^16^ ^]^ (Table S1, Supporting Information). To date, how exactly elasticity of nanoformulations affect their biological performance is still unclear or even debatable. For example, to the best of our knowledge, few studies have investigated the relationship between nanoformulation elasticity and their BBB permeability,^[^ ^17^ ^]^ given that BBB is one of the most challenging barrier for nanoformulation‐based brain diseases therapy.^[^ ^18^ ^]^ Most of the in vitro studies have claimed that nanoformulations with higher elasticity are more favorable for internalization by cancer cells,^[^ ^11b^ ^]^ while others demonstrated that lower elastic nanoformulations displayed more potent tumor cell uptake capacity.^[^ ^19^ ^]^ Similarly, in vivo studies also showed contradictory results, one report showed the softest nanoformulations had higher tumor accumulation,^[^ ^11b^ ^]^ whereas another reported the semi‐elastic nanoformulations exerted higher tumor accumulation than their stiffest or softest counterpart.^[^ ^11a^ ^]^ Additionally, the in vitro tumor model penetration exercise are also inconsistent between studies.^[^ ^11^, ^20^ ^]^ As for cellular internalization, tumor accumulation and penetration, results on nanoformulations’ biodistribution are also mixed. Mitragotri's group found that soft particles showed more accumulation in lungs than the hard ones 12 h post injection, while little difference displayed in liver, spleen, kidney, heart, brain.^[^ ^11e^ ^]^ However, another research demonstrated that hard nanoformulations accumulated more in spleen 48 h after injection, while similar accumulation were observed in other organs.^[^ ^21^ ^]^ These unclear performance or inconsistencies suggest much more in‐depth investigations are required in regards to “nanoformulation elasticity‐biological performance”. In particular for brain tumor targeting, how the mechanical properties of nanoformulations affect their ultimate brain destinations, to our best of knowledge, has never been reported.

**Figure 1:**
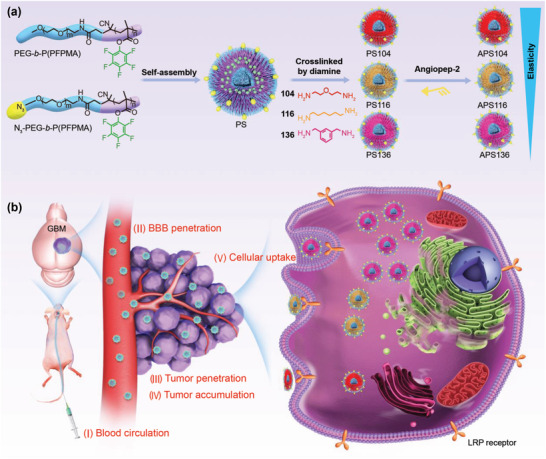
Schematic illustration. a) The preparation of polymersomes with varying elasticity by tuning their membrane‐diamine‐crosslinkers. b) Representation of biological performance including blood circulation, BBB penetration, tumor penetration, tumor accumulation, and cellular uptake.

Here, we developed a library of membrane‐crosslinked polymersomes with varying elasticity to investigate their brain targeting destinies. The elasticity of polymersomes was tuned with different diamine crosslinkers (**Figure** **1**a). After surface decoration with a frequently utilized brain tumor targeting peptide ligand, angiopep‐2, which is capable of penetrating BBB and targeting brain tumor,^[^ ^22^ ^]^ we assessed the biological performance of these brain targeting polymersomes including cellular uptake, BBB permeation, tumor penetration, blood circulation and brain tumor accumulation to systematically unravel the underlying mechanisms of their biological destinations (Figure 1b). To the best of our knowledge, we are the first to investigate the relationship between nanoformulation elasticity and biological performances in the brain.

## 2 Results and Discussions

**Figure 2:**
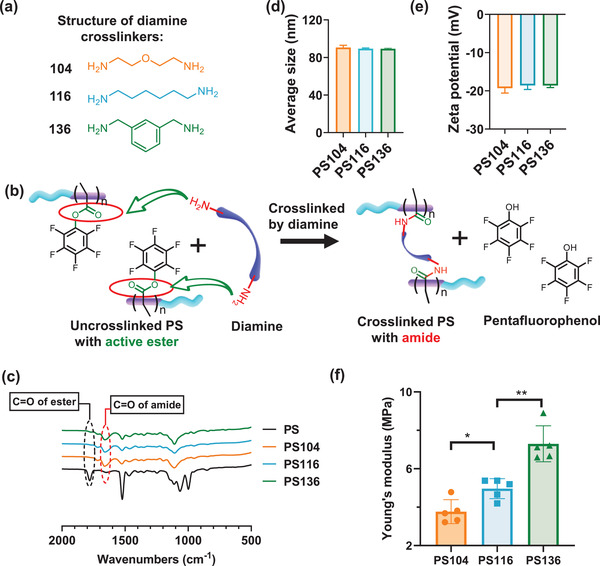
Preparation and physical characterization of membrane‐crosslinked polymersomes with varying elasticity. a) Different diamine crosslinkers used in this study. b) Schematic illustration of the crosslinking progress of polymersome. c) FT‐IR spectra of membrane‐crosslinked polymersomes with different diamine crosslinkers. d) Particle size and e) zeta potential of different diamine crosslinked polymersomes. Data are presented as mean ± SD (*n* = 3). f) Young's modulus of polymersomes crosslinked by different diamines determined by AFM. Data are presented as mean ± SD (*n* = 5). These data were analyzed using one‐way *t*‐test, *p < 0.05, **p < 0.01.

In this study, polymersome was selected as a model nanoformulation due to its versatility in loading a variety of different drugs.^[^ ^23^ ^]^ The precursor polymersomes were prepared by self‐assembly from poly(ethylene glycol)‐*block*‐poly(pentafluorophenyl methacrylate) block copolymers (PEG‐*b*‐P(PFPMA)), which was synthesized by reversible addition fragmentation chain transfer (RAFT) polymerization (Figure S1, Supporting Information). The molecular weight of PEG‐*b*‐P(PFPMA) is at around 2–5 kDa by ^1^H NMR spectrum based calculation (Figure S2, Supporting Information), which is considered an ideal hydrophilic‐hydrophobic ratio for amphiphilic diblock polymers self‐assembly into polymersome.^[^ ^24^ ^]^ Notably, PEG‐*b*‐P(PFPMA) derived polymersomes displayed small particle size (85 nm in diameter) and narrow size distribution (polydispersity index, PDI = 0.25) (Table S2, Supporting Information). The membrane of the precursor structure can be permanently fixed by a crosslinking reaction of the hydrophobic pentafluorophenyl ester moieties with diamine crosslinkers (oxyethylene bis(amine) (MW = 104), hexamethylenediamine (MW = 116), bis(aminomethyl)benzene (MW = 136)) (**Figure** **2**a,b). The crosslinking reaction was confirmed by Fourier transform infrared spectroscopy (FT‐IR) analyses (Figure 2c), in which absorption of ester C═O groups at 1790 cm^−1^ in precursor disappeared, while the absorption of amide C═O groups at 1632 cm^−1^ was observed after diamine crosslinking. The disappearance of the activated carbonyl group in contrast with the appearance of the amide carbonyl group suggested that the hydrophobic pentafluorophenyl ester moieties were completely converted to amide groups after crosslinking. Besides, this crosslinking was also verified by ^19^F NMR analysis (Figure S3, Supporting Information), in which the signals of fluorine showed a significant shift after crosslinking. Note that morphologically, these nanoformulations kept their spherical vesicular structure after crosslinking (Figure S4, Supporting Information). More importantly, these crosslinked polymersomes showed similar particle size (around 90 nm in diameter) (Figure 2d and Table S2, Supporting Information) and zeta potential (around −19 mV) (Figure 2e), which means their relative biological performances independent of size or surface charge. It was noted that these crosslinked polymersomes showed similar particle size in comparison to the uncrosslinked parent nanoformulations (Table S2, Supporting Information). This phenomenon could be ascribed to the crosslinking process only happened on the hydrophobic membrane median layer via intramolecular reaction, without changing the shape or nanostructure of these polymersomes. In addition, these crosslinked polymersomes are remarkably stable for 9 days (Figure S5, Supporting Information). Next, the mechanical properties of these crosslinked polymersomes were characterized using atomic force microscopy (AFM). These results showed that polymersomes containing the benzyl crosslinkers demonstrated higher Young's modulus than polymersomes containing the ethylene glycol containing crosslinkers (Figure 2f). The elasticity of these polymersomes was closely related to the rigidity of their incorporated crosslinkers, such that the benzyl containing crosslinker generated less elastic polymersomes than alkene containing polymersomes, which in turn, were less elastic than ethylene glycol containing polymersomes. Due to the high rigidity of the pentafluorophenyl groups, the uncrosslinked polymersomes exerted higher elasticity than their crosslinked counterparts. (Table S2, Supporting Information). For further biological evaluation, we modified these diamine (oxyethylene bis(amine) (MW = 104), hexamethylenediamine (MW = 116), bis(aminomethyl)benzene (MW = 136)) crosslinked polymersomes with angiopep‐2 and denoted as APS104, APS116, APS136, respectively.

**Figure 3:**
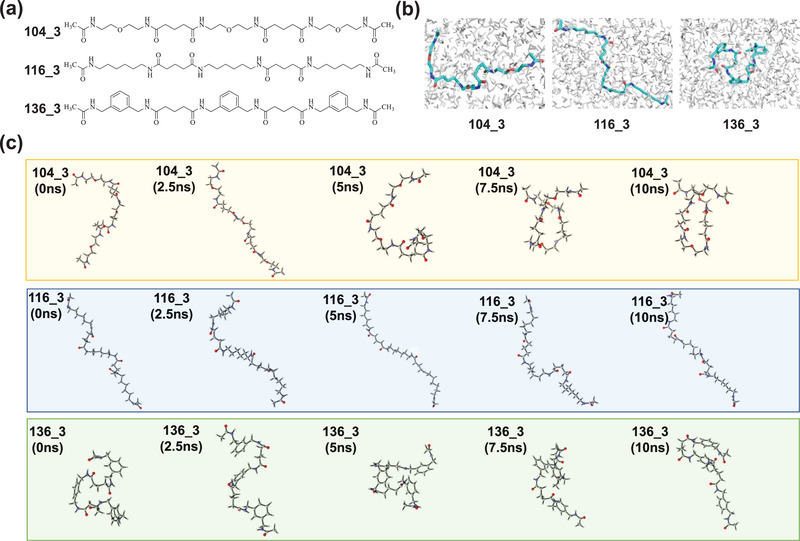
Theoretical calculations and simulations. a) Structures of 104_3, 116_3, and 136_3. b) Structures of 104_3, 116_3, and 136_3 in the water box. c) Conformational changes of 104_3, 116_3, and 136_3 in molecular dynamics simulation.

Next, we performed theoretical calculations and simulations. Regarding the crosslinking reaction product of the hydrophobic pentafluorophenyl ester moieties with diamine crosslinkers (104, 116, 136). We simplify the product model with three structural units, which is also the simplest structure that can represent multiple chains (**Figure** **3**a). The difference among the polymersomes is the diamine crosslinkers. Therefore, the hydrophobic pentafluorophenyl ester moieties and diamine crosslinkers form a simplified chain structure, and its rigidity or flexibility determines the stiff or soft of nanoformulations. In order to study the elasticity of their structures, we optimized the models of 104_3, 116_3, and 136_3 by quantum mechanics, and put the optimized structure in the water box to perform molecular dynamics simulation, as shown in Figure 3b. We extracted the dynamic structures of the three molecules (104_3, 116_3, 136_3) at 0, 2.5, 5, 7.5, and 10 ns in the molecular dynamics simulation, as shown in Figure 3c. We can see the changes in the skeleton structure of the three molecules. The skeleton of 104_3 has experienced a state from molecular stretching to molecular folding, and the molecular skeleton has a larger fluctuation range; the skeleton of 116_3 has relatively less volatility compared to 104_3; while 136_3 has basically been in a state of molecular folding, the molecular skeleton fluctuation range is the smallest among the three molecules. Radius of gyration (*R*_g_) analysis can confirm this statement, as shown in Figure S6 (Supporting Information). At the same time, we carried out root‐mean‐square deviation (RMSD) analysis on the results of molecular dynamics simulation. RMSD can reflect the fluctuation of the molecular skeleton structure, and then can show whether the overall structure of the molecule is rigid or flexible.^[^ ^25^ ^]^ The results are shown in Figure S7 (Supporting Information). The RMSD peaks of the three structures 104_3, 116_3, and 136_3 are about 7.7, 7.6, and 7.5, respectively. Therefore, the degree of deformation of the three molecules varies from small to large is 136_3, 116_3, and 104_3, and it can be inferred that the deformation of the polymersomes from small to large is PS136, PS116, and PS104. The result was consistent with the Young's modulus of polymersomes in Figure 2f.

**Figure 4:**
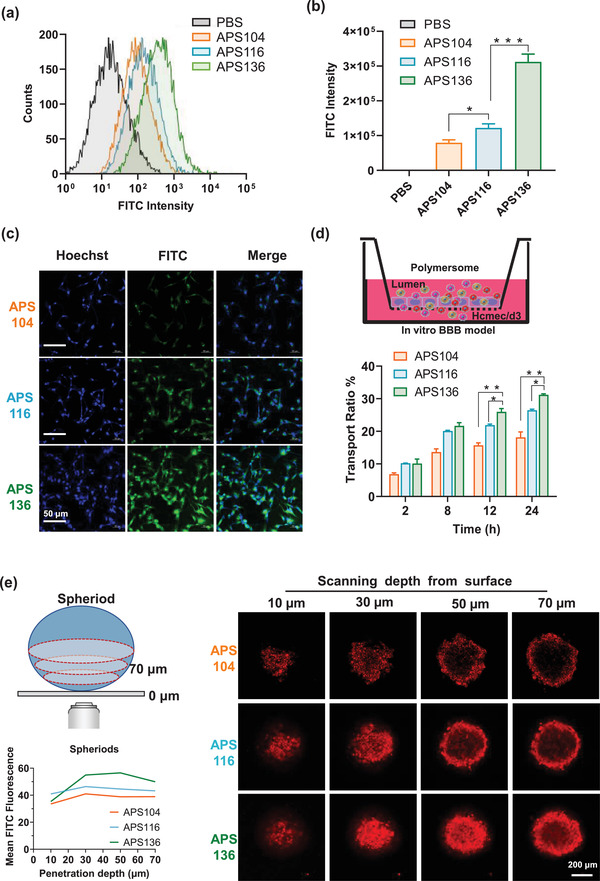
In vitro biological performance of polymersomes with different elasticity. a) Cellular uptake of different elastic polymersomes in U87MG cells determined by flow cytometry. b) Cell lysis as determined by FITC fluorescence intensity after treatment with different polymersomes incubated with U87MG for 8 h. c) CLSM images of U87MG cells incubated with polymersomes for 8 h. Scale bar = 50 µm. d) An in vitro model of the BBB and accumulative transport ratio of polymersomes across the in vitro model of the BBB at different time points. Data are presented as mean ± SD (*n* = 3). e) Penetration of polymersomes into U87MG multicellular spheroids. Z‐stack images were obtained starting from the top and progressing into the spheroid core at intervals of 20 µm. Scale bar = 200 µm.

Then, these brain‐tumor targeted polymersomes were utilized to study the effects of mechanical properties on specific (receptor‐mediated) nanoformulation cellular uptake. For better trafficking and monitoring, these polymersomes were labeled by FITC dye. Stiff polymersomes showed better cellular uptake than the soft counterparts as shown by flow cytometry assay, after incubation with U87 glioblastoma cells for 8 h (**Figure** **4**a). These results were further confirmed by cell lysis assay and confocal laser scanning microscope (CLSM) imaging (Figure 4b,c), that the polymersomes with less elasticity showed better cellular uptake. These results were consistent with previous results that tumor cells are preferentially take up hard nanoformulations.^[^ ^19^, ^20^, ^26^ ^]^ This phenomenon maybe resulted from the possibility that the stiff polymersomes kept their spheroids shape during both binding to the cell membrane and internalization, whereas the soft polymersomes displayed deformations during both processes.^[^ ^19^ ^]^ The deformation has reduced the polymersomes ability for cell binding and require more energy for cell internalization.

Subsequently, an in vitro BBB model was established to investigate the influence of polymersomes elasticity in BBB penetration.^[^ ^27^ ^]^ The BBB is the most stubborn challenge for brain tumor therapy,^[^ ^18^ ^]^ which highlights the importance of understanding the mechanisms behind the nanoformulation‐BBB interaction. As shown in Figure 4d, in line with in vitro cellular uptake, the stiffest polymersomes also showed the most potent BBB penetration. Considering that BBB penetration is mediated by receptor‐mediated transcytosis, the stiffest polymersomes not only showed better cell endocytosis but also exerted more potent transcytosis. We then used U87MG multicellular spheroids to evaluate the penetration behavior of these polymersomes in tumors (Figure 4e). For soft polymersomes, fluorescence mostly occurred on the periphery of the multicellular spheroids at a scanning depth of 50 µm. In contrast, the penetration capability of the hard polymersomes was significantly higher, as fluorescence could be clearly observed even at a scanning depth of 70 µm inside the multicellular spheroids, demonstrating the superior permeability of the hard polymersomes.

**Figure 5:**
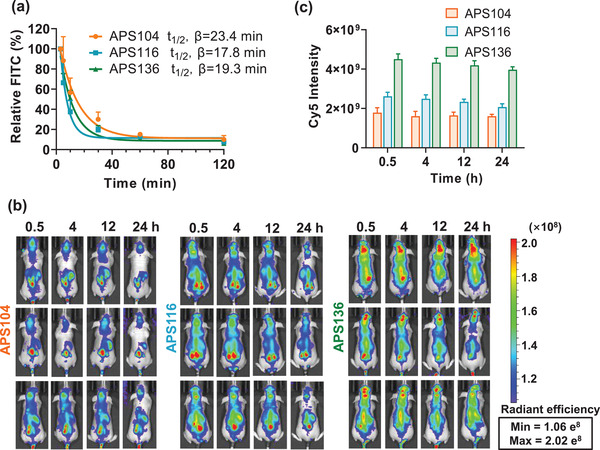
In vivo biological performance of polymersomes with different elasticity in a mouse model of brain tumor. a) In vivo pharmacokinetics of polymersomes with different elasticity in mice. b) Fluorescence images of nude mice bearing orthotopic U87MG‐luc human glioblastoma. c) Quantification of fluorescence intensity of nude mice bearing orthotopic U87MG‐Luc human glioblastoma at different time points following i.v. injection of different elastic polymersomes. Data are presented as mean ± SD (*n* = 3).

After in vitro biological assessment, in vivo comparisons were subsequently investigated. First, the role of polymersome elasticity on circulation time was investigated by intravenously injecting an identical quantity of polymersomes into mice. The softest polymersomes showed partially increased circulation time in blood (Figure 5a). Then, the role of polymersome elasticity on tumor accumulation was assessed in nude mice bearing orthotopic luciferase‐tagged U87MG cells following tail vein injection. In this study, U87MG‐luc cells were used to enable tumors to be monitored in vivo by bioluminescence. The results showed that the mice treated with the stiffest polymersomes showed significantly greater accumulation in brain tumor as judged by increased Cy5 fluorescence in the tumor area (**Figure** **5**b,c). Though the soft polymersomes showed partially increased circulation time in blood, the polymersomes with the highest stiffness exerted the most brain tumor accumulation. This observation can be attributed to the fact that the combined effects of cellular uptake, BBB permeation and tumor penetration play a dominant role for in vivo brain tumor accumulation in contrast to blood circulation.

## 3 Conclusion

In summary, we prepared a library of membrane‐crosslinked polymersomes that exhibited different elasticity. By tuning the crosslinker, we demonstrated that polymersomes elasticity influences brain tumor targeting. In comparison with soft polymersomes, stiff polymersomes showed superior brain tumor cell uptake ability, potent in vitro BBB crossing ability, excellent tumor penetration and higher orthotopic brain tumor accumulation, despite comparable particle size, zeta potential and morphology. Hence, this study offers fundamental insights into how the mechanical properties of nanoformulation influence their biological performance which provides guidance for the future design of mechanically engineered nanoformulations for enhanced drug delivery for the treatment of brain tumors or potentially neurodegenerative disease.

## Conflict of Interest

The authors declare no conflict of interest.

## Supporting information

Supporting Information

## Data Availability

The data that support the findings of this study are available from the corresponding author upon reasonable request.
