# Supplementary material for: Tuning the Elasticity of Polymersomes for Brain Tumor Targeting
Source: Adv Sci (Weinh). 2021 Aug 22;8(20):2102001. doi: 10.1002/advs.202102001 (PMC8529491; doi:10.1002/advs.202102001)
Supplement: Supplementary file 1 — Supporting Information [file ADVS-8-2102001-s001.pdf]

© 2021 Wiley-VCH GmbH

ADVANCED SCIENCE
Open Access

Supporting Information

for *Adv. Sci.,* DOI: 10.1002/advs.202102001

Tuning the Elasticity of Polymersomes for Brain Tumor Targeting

*Meng Zheng, Qiuli Du, Xin Wang, Yuan Zhou, Jia Li, Xue Xia, Yiqing Lu, Jinlong Yin, Yan Zou, Jong Bae Park and Bingyang Shi**

Supporting information

Tuning the Elasticity of Polymersomes for Brain Tumor Targeting

*Meng Zheng†, Qiuli Du†, Xin Wang, Yuan Zhou, Jia Li, Xue Xia, Yiqing Lu, Jinlong Yin, Yan Zou, Jong Bae Park and Bingyang Shi**

Dr. M. Zheng, Q. L. Du, Dr. X. Wang, Y. Zhou, Dr. J. Li, Dr. X. Xia, Prof. Y. Lu, Prof. J. Yin, Dr. Y. Zou, Prof. B. Shi
Henan and Macquarie University Joint Centre for Biomedical Innovation
School of Life Sciences, Henan University, Kaifeng, 475004, China
Henan Key Laboratory of Brain Targeted Bio-nanomedicine
School of Life Sciences & School of Pharmacy, Henan University, Kaifeng, 475004, China
E-mail: bs@henu.edu.cn

Dr. Y. Zou, Prof. B. Shi
Department of Biomedical Sciences, Faculty of Medicine & Health Sciences
Macquarie University, Sydney, NSW 2109, Australia

Prof. Y. Lu
School of Engineering, Faculty of Science and Engineering, Macquarie University, Sydney, NSW 2109, Australia

Prof. J. Park
Department of Cancer Biomedical Science, Graduate School of Cancer Science and Policy, National Cancer Center, Goyang, 10408 South Korea

† These authors contributed equally to this work

Experimental Section

Materials. Pentafluorophenyl methacrylate (PFPMA, 97%, Aladdin), 4-cyanopentanoic acid dithionaphthalenoate (CPADN) (Aladdin), 2,2'-azobis(2-methylpropionitrile) (AIBN, 99%, Aladdin), *N, N'*-dicyclohexyl carbodiimide (DCC, 99%, Alfa Aesar), *N*-hydroxysuccinimide (NHS, 98%, Alfa Aesar), oxyethylene bis(amine) (98%, Aladdin), hexamethylenediamine (99.5%, Aladdin), bis(aminomethyl) benzene (99%, J&K), 1-amino-3,6,9-trioxaundecanyl-11-ol (99%, Aladdin), fluorescein-5-maleimide (97%, Aladdin) were used as received. Azide-poly(ethylene glycol)-amine (N_3-PEG-NH_2, 2 kDa, 95%) was purchased from Ponsure Biotechnology (Shanghai, China). Methoxy-poly(ethylene glycol)-amine (MeO-PEG-NH_2, 2 kDa, 95%) was obtained from Jenkem (Beijing, China). Cy5-amine (Cy5-NH_2) and DBCO-NHS were supplied by Ruixi Biological Technology Co., Ltd. (Xi'an, China). Angiopep-2 peptide terminated with amine group (95%) was bought from China Peptides Co., Lt. (Shanghai, China).

Synthesis of MeO/N_3-PEG-CPADN. A 50 mL reaction flask was purged with nitrogen for 30 min. The CPADN (62.55 mg, 225 μmol) and NHS (28.47 mg, 247.5 μmol) were dissolved in unhydrous tetrahydrofuran (THF), then the mixture was cooled down to 0 °C. DCC (50.99 mg, 247.5 μmol) dissolved in 10 mL THF was dropped into the cold CPADN/NHS mixture. After 12h reaction, MeO-PEG-NH_2 (100 mg, 50 μmol) or N_3-PEG-NH_2 (100 mg, 50 μmol) dissolved in 5 mL unhydrous THF was added and stirred overnight at room temperature. Then, the reaction solution was filtered, precipitated in cold diethyl ether for 3 times, and subsequently dried under vacuum.

Synthesis of MeO/N_3-PEG-b-P(PFPMA). MeO-PEG-CPADN or N_3- PEG-CPADN (22.2 mg, 10 μmol), PFPMA (120.96 mg, 480 μmol) and AIBN (0.25 mg, 1.5 μmol) were dissolved in unhydrous dioxane and sealed in a flask under the protection of nitrogen. The reaction was proceeded under 65 °C for 48 h. The solution was cooled down to r.t. and precipitated 3 times

in cold hexane. After drying in vacuum, the pink products of MeO/N_3-PEG-b-P(PFPMA) were obtained. The molecular weight of MeO/N_3-PEG-*b*-P(PFPMA) were 2-5 kDa by ^{1}H NMR spectrum based calculation. For polymer fluorescence labeling, fluorescein-5-maleimide and Cy5-NH_2 were utilized. As for fluorescein labeling, PEG-*b*-P(PFPMA) (40 mg, 5.7 μmol), AIBN (0.15 mg, 0.9 μmol) were dissolved in 2 mL dry dioxane, then fluorescein-5-maleimide (4.9 mg, 11.4 μmol) dissolved in 1 mL dimethyl sulfoxide (DMSO) was added. The mixture was sealed in a flask under the protection of nitrogen and stirred under 65 °C for 48 h. For Cy5 labled PEG-*b*-P(PFPMA), the Cy5-NH_2 was graft onto the PEG-*b*-P(PFPMA) by active ester reaction. Briefly, PEG-*b*-P(PFPMA) (40 mg, 5.7 μmol) was dissolved in 1 mL dioxane and the DMSO solution of Cy5-NH_2 (4 mg/mL, 1 mL) was added. The reaction was conducted at 60 °C for 24 h. All the fluorescence labeling reaction mixtures were dialyzed against double distilled water for 2 days. After dialysis, the solutions were freezing dried.

Synthesis of Angiopep-2-DBCO. First, Angiopep-2 (50 mg, 20.8 μmol) and DBCO-NHS (10.0 mg, 24.87 μmol) were dissolved in 2 mL DMSO and added into a 10 mL reaction tube. After stirring at room temperature overnight, the solution was dialyzed with 2 kDa MWCO dialysis bag against water for 2 days. The product Ang-DBCO was acquired by freeze drying.

Formation and crosslinking of polymersomes. The PEG-*b*-P(PFPMA) (10 mg, 1.4 μmol polymer or 28 μmol reactive ester) was dissolved in 1 mL THF, and then dropped into 10 mL anhydrous DMSO. After 3 h stirring to remove the THF, the uncrosslinked polymersomes were formed in DMSO. For crosslinking, the triethylamine (19.39 μL, 140 μmol) and different diaimne crosslinkers (14 μmol) were added. After 24 h stirring at 60 ℃, excess of 1-amino-3,6,9-trioxaund ecanyl-11-ol (54.04 mg, 280 μmol) was added and stirred for additional 24 h at 60 ℃ to remove unreacted pentafluorophenol ester. Next, the reaction mixture was purified by dialysis and the crosslinked polymersomes (PS) were obtained. As for Angiopep-2 decorated polymersomes (APS), the crosslinked polymersomes formed by

MeO-PEG-*b*-P(PFPMA)/N_3-PEG-*b*-P(PFPMA) mixture at the mole ratio of 4:1 were reacted with Angiopep-2-DBCO in aqueous solution for 2 days. Next, the reaction mixture was purified by dialysis and the Angiopep-2 decorated crosslinked polymersomes were obtained.

Characterization of polymersomes. The hydrodynamic size, polydispersity index (PDI) and zeta potential of polymersomes were measured at 25 °C with the Zetasizer Nano ZS instrument. As for the stability test of polymersomes, the freshly prepared polymersomes solution were stored in EP tubes at room temperature. The size was measured every other day until day 9. The size presented in the main text body was measured by DLS based on intensity and was represented as d50. The FT-IR spectra of crosslinked polymersomes were measured by Fourier transform infrared spectrometer (Bruker, VERTEX70). Morphologies of polymersomes were examined using a transmission electron microscope (TEM, JEM-2100, JEOL, Tokyo, Japan) at an acceleration voltage of 200 kV and a beam current of 40 μA. Each sample was placed on 3 mm-mesh copper grids and stained with uranyl acetate solution (1% w/v) for 15 min.

Elasticity measurement. AFM force measurements were conducted using a Bio-FastScan scanning probe microscope (Bruker) in PeakForce QNM imaging mode. The crosslinked polymersomes suspensions were dropped onto a freshly cleaved mica surface and air-dried at room temperature for 1 h. The samples were imaged at a scan rate of 1.4 Hz. The data of polymersomes obtained from AFM were processed by the software NanoScope Analysis (Bruker), and the Young's modulus was calculated by processing the images of polymersomes in PeakForce QNM mode (n= 5).

Computational details. The DFT calculations were performed with the Gaussian 16 program package.[1] The geometry optimizations of minima and transition states involved were carried out at the B3LYP-D3 level of theory[2] with the 6-31G(d) basis set. The vibrational frequencies were computed at the same level to check whether each optimized

structure is an energy minimum or a transition state and to evaluate its zero-point vibration energy (ZPVE) and thermal corrections at 298 K. The conformer with lowest energy was presented in the supporting information. The structures were solvated in a water box using AmberTools 18.[3] Classical MD was performed using Amber 18 on the compound structures for 10 ns in water box. The molecular models constructed in the water box was used for MD simulations with RESP [4] charges assigned on compound structures. General Amber Force Field (GAFF)[5] was used for compound structures. In the water, the system was minimized for 20000 steps, and gradually heated to 300 K and then equilibrated for 10ns under constant T and V. Five snapshots were taken in the 10 ns with 2.5 ns interval to form an ensemble of compound structures.

Flow cytometry assay. The U87MG cells were seeded in 12-well plate (5.0×10^5 cells/well) and cultured overnight with DMEM medium containing 10% fetal bovine serum (FBS). The cells were incubated with different FITC labled APS at 37 °C at the same concentration. After 8 h incubation, the cells were washed twice with PBS and digested by trypsin. The suspensions were centrifuged at 1000 × g for 3 min, then resuspended in 400 µL PBS. All samples were analyzed by flow cytometry (FACS Calibur, BD Bioscience, USA). For cell lysis, the U87MG cells were washed and lysed at 4 ℃ after 8 h incubation with FITC labled APS. The fluorescence of cell lysis solution was measured by microplate reader (Ex = 492 nm, Em =525 nm).

Confocal laser scanning microscopy assay. To visualize the cell uptake of different APS, U87MG cells were cultured on microscope plate (1×10^5 cells/well) overnight. Then cells were incubated with 100 µL different crosslinked FITC labled APS at 37 °C for 8 h. The cells were rinsed with PBS buffer for three times, fixed with 4% paraformaldehyde solution for 15min, and then washed with PBS two times. The cell nuclei were stained with Hoechst (10

μg/mL) for 10 min and washed with PBS for three times. The fluorescence images were obtained using a confocal microscope (Zeiss 880).

BBB permeation via an in vitro BBB model. To compare the BBB permeation ability of different elastic polymersomes, an *in vitro* BBB model was established by seeding a monolayer immortalized human brain microvascular endothelial cells hCMEC/D3 (5×10^4 cells/well) on the upper chamber of culture inserts (Corning, NY, USA). The lower chamber was filled with 800 μL medium. The culture medium was refreshed every 2 days. When the trans-endothelial electrical resistance (TEER) value of the monolayer hCMEC/D3 was higher than 200 Ω cm^2, the transwells could be used for study. For transcytosis study, the cell culture medium in upper chamber was replaced with 450 μL serum free medium, and then 50 μL FITC labled APS were added. The transwells were then put on a shaker (50 rpm). At 2, 8, 12 and 24 h time point, 200 μL of aliquots were collected from the basolateral compartment and replaced with same volume of fresh medium. At the end of the experiment, the TEER was measured again to monitor the integrity of the hCMEC/D3 cells monolayers. Finally, the transport ratio (%) of APS was measured by determining the fluorescence of the samples by microplate reader (Ex = 492 nm, Em =525 nm).

Penetration in U87MG multicellular spheroids. To observe the penetration abilities of APS in multicellular spheroids, the U87MG cells were seeded in 96-well plate (PrimeSurface ™, MS-9096U) (5×10^3 cells/well). After 48 h when the diameter of multicellular spheroids reached about 500 μm in diameter, the spheroids were cultured with Cy5 labled APS for 6 h. The pellets were washed three times with PBS and fixed with 4% paraformaldehyde solution for 30 min and then transferred into confocal dishes. The fluorescence of Cy5 was detected using Z-stack imaging, with 10 μm intervals from the top of the spheroids to the middle, using a Zeiss 880 confocal microscope.

Pharmacokinetics study. FITC labled APS in 200 µL of PBS were intravenously injected into Balb/c mice *via* the tail vein (n = 3). At prescribed time points post injection, ca. ~50 µL of blood was taken out from the eye socket. The blood samples were centrifugated (3000 rpm, 30 min) immediately, and the FITC level in the supernatant was determined by detecting the fluorescence intensity using a microplate reader (Ex = 492 nm, Em = 525 nm). The half-lives were calculated by fitting the experimental data using Software Origin 2018b exponential decay 2 model: $y = A_1 \times \exp(-x/t_1) + A_2 \times \exp(-x/t_2) + y_0$, and then taking $t_{1/2,\beta} = 0.693\times t_2$.

In vivo imaging. To evaluate the *in vivo* tumor targeting ability, Cy5 labled APSs were injected intravenously to U87MG-luc orthotopic tumor-bearing nude mice and monitored at different time points by using the Lumina IVIS III imaging system (Ex = 620 nm; Em = 670 nm).

Statistical Analysis. Differences between two groups were assessed using unpaired t tests. Data were analyzed by GraphPad Prism software. All data were expressed as mean ± SD. Data analyses were performed with t-test. $p < 0.05$ was considered statistically significant.

Table S1. Possible factors impact the elasticity of nanoformulations. [6]

Nanoformations	Methods to tune the elasticity
Hydrogel particles	● The polymer crosslinking density[7]<br>● The polymer volume fraction[8]
Nanocupsules or liposomes	● Choice of material[9]<br>● Thickness or number of layers[10]<br>● Extent of layer crosslinking[11]<br>● Encapsulated components[12]

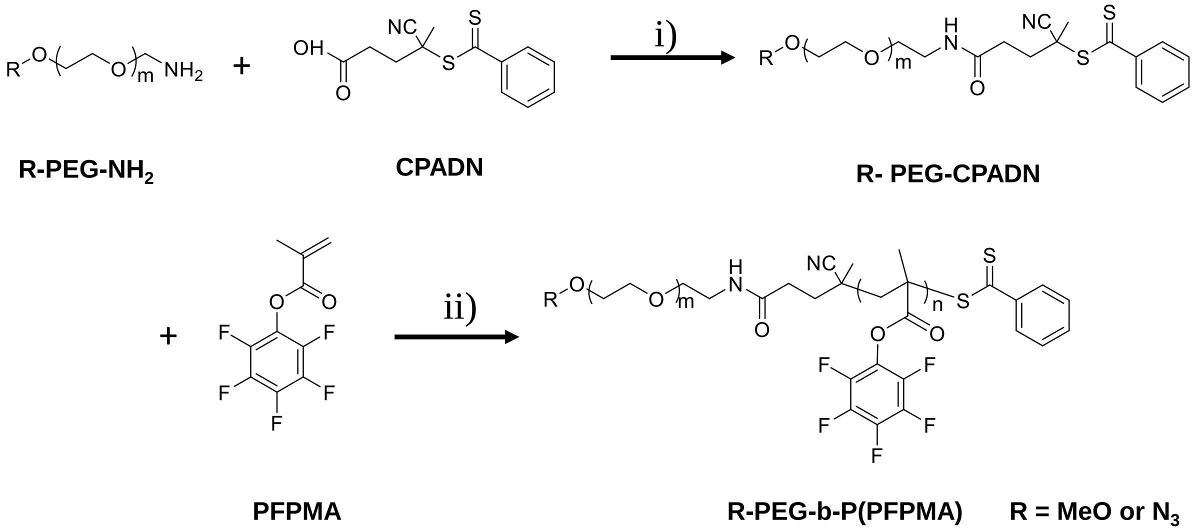

Figure S1. Synthetic pathway for MeO(N_3)-PEG-*b*-P(PFPMA). Conditions: (i) DCC/NHS, THF, r.t., 24h. (ii) AIBN initiator, 1,4-dioxane, 65 °C, 48 h.

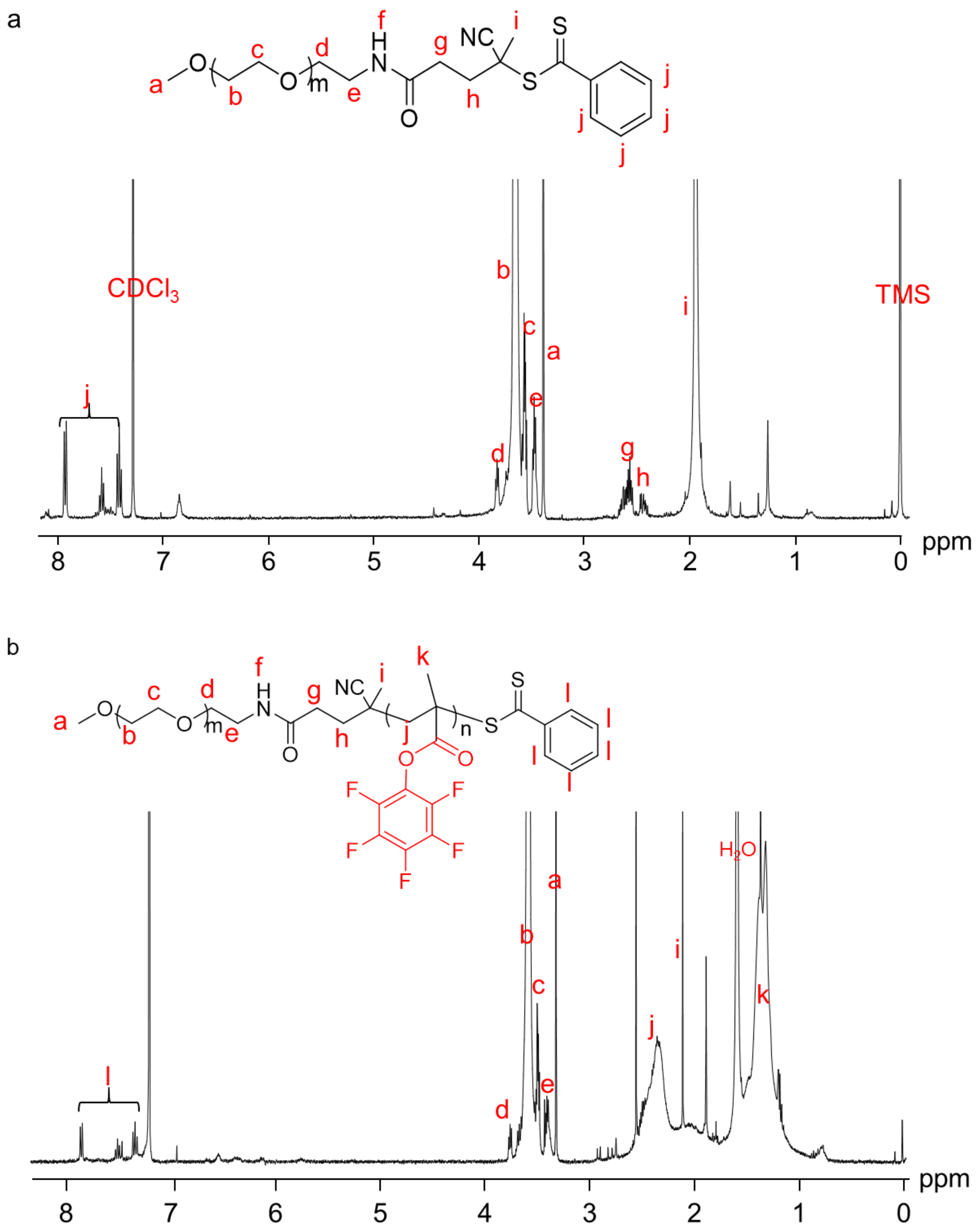

Figure S2. [1]*H* NMR spectra (400 MHz) of (a) MeO-PEG-CPADN ($CDCl_3$) and (b) MeO-PEG-b-P(PFPMA) ($CDCl_3$).

Table S2. Size, polydispersity index (PDI), zeta potential and Young's Modulus of uncroslinked or crosslinked polymersomes.

Polymersomes	Uncrosslinked	PS104	PS116	PS136
Size (nm)	85.0±1.40	90.6±2.43	89.3±0.75	89.3±0.59
PDI	0.25±0.01	0.18±0.01	0.21±0.02	0.18±0.01
Zeta potential (mV)	-19.0±0.29	-19.3±1.27	-18.6±1.06	-18.6±0.50
Young's Modulus (MPa)	11.08±1.35	3.77±0.62	4.96±0.52	7.30±0.94

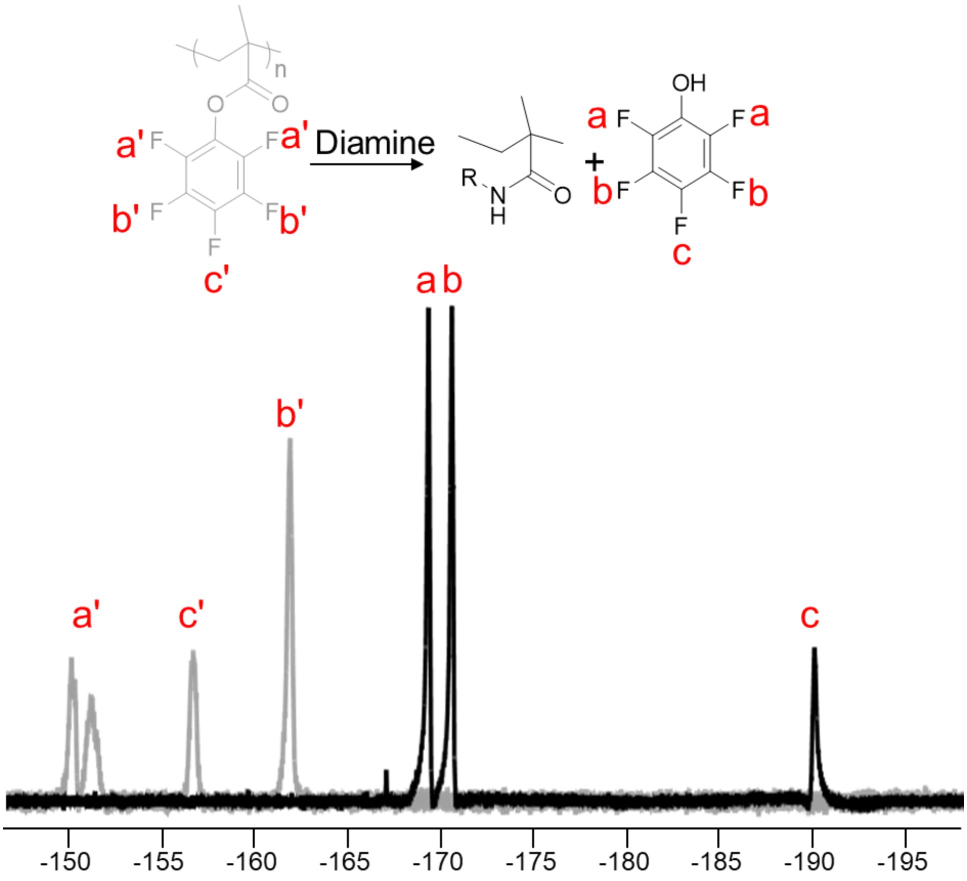

Figure S3. ^{19}F NMR spectrum (400 MHz) of MeO-PEG-*b*-P(PFPMA) before and after crosslinking ($CDCl_3$).

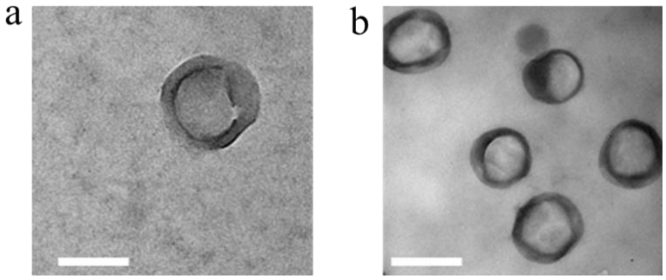

Figure S4. TEM images of polymersomes (a) before and (b) after crosslinking (Scale bar=100 nm).

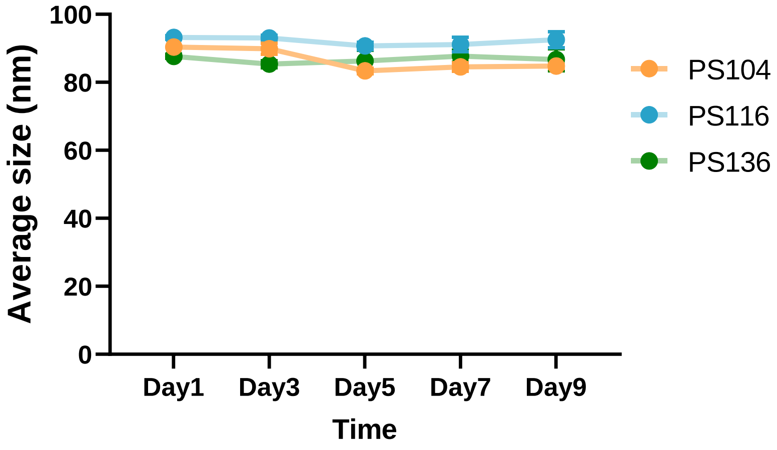

Figure S5. Stability of polymersomes after crosslinking.

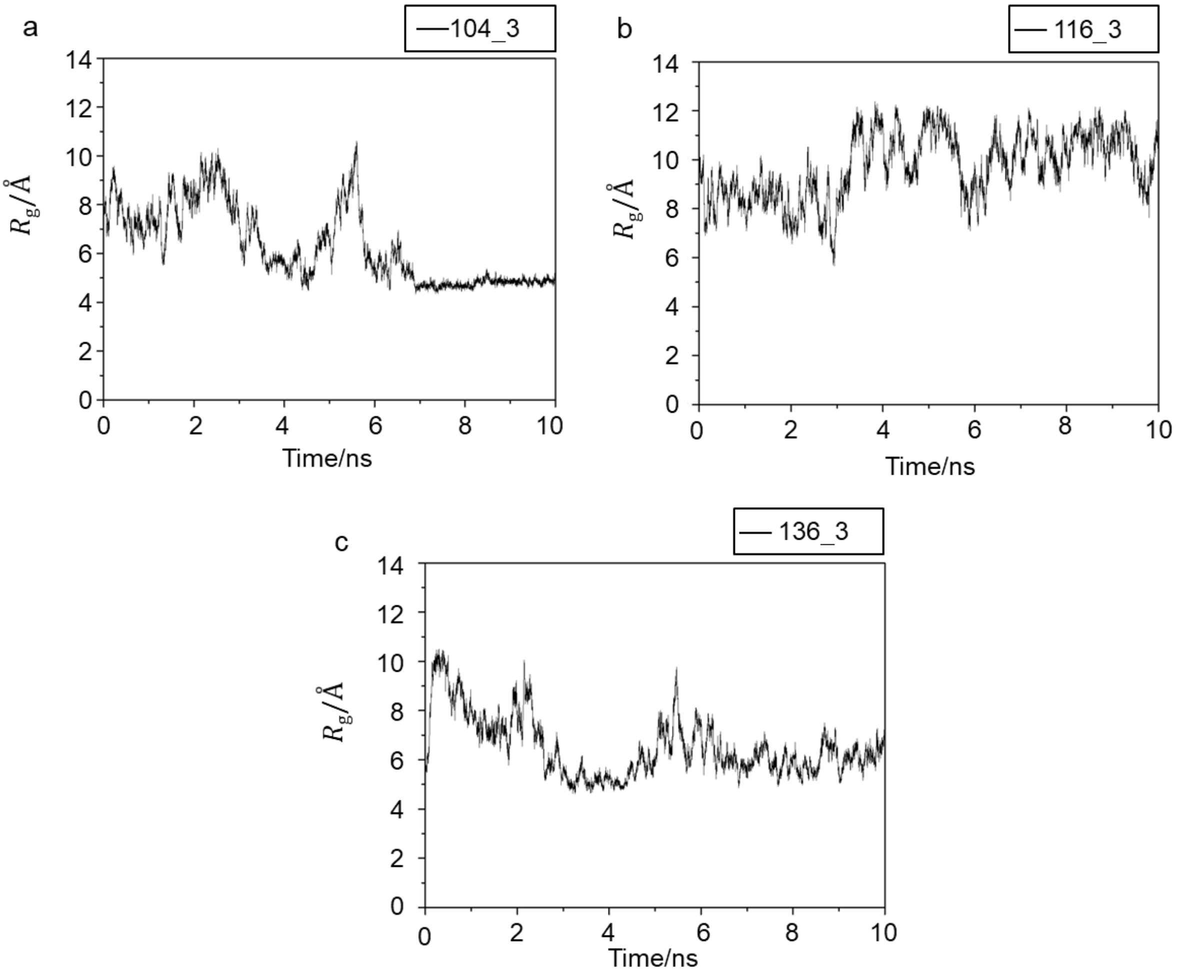

Figure S6. Radius of gyration (R_g) of (a) 104_3, (b) 116_3 and (c) 136_3 for 10 ns of MD.

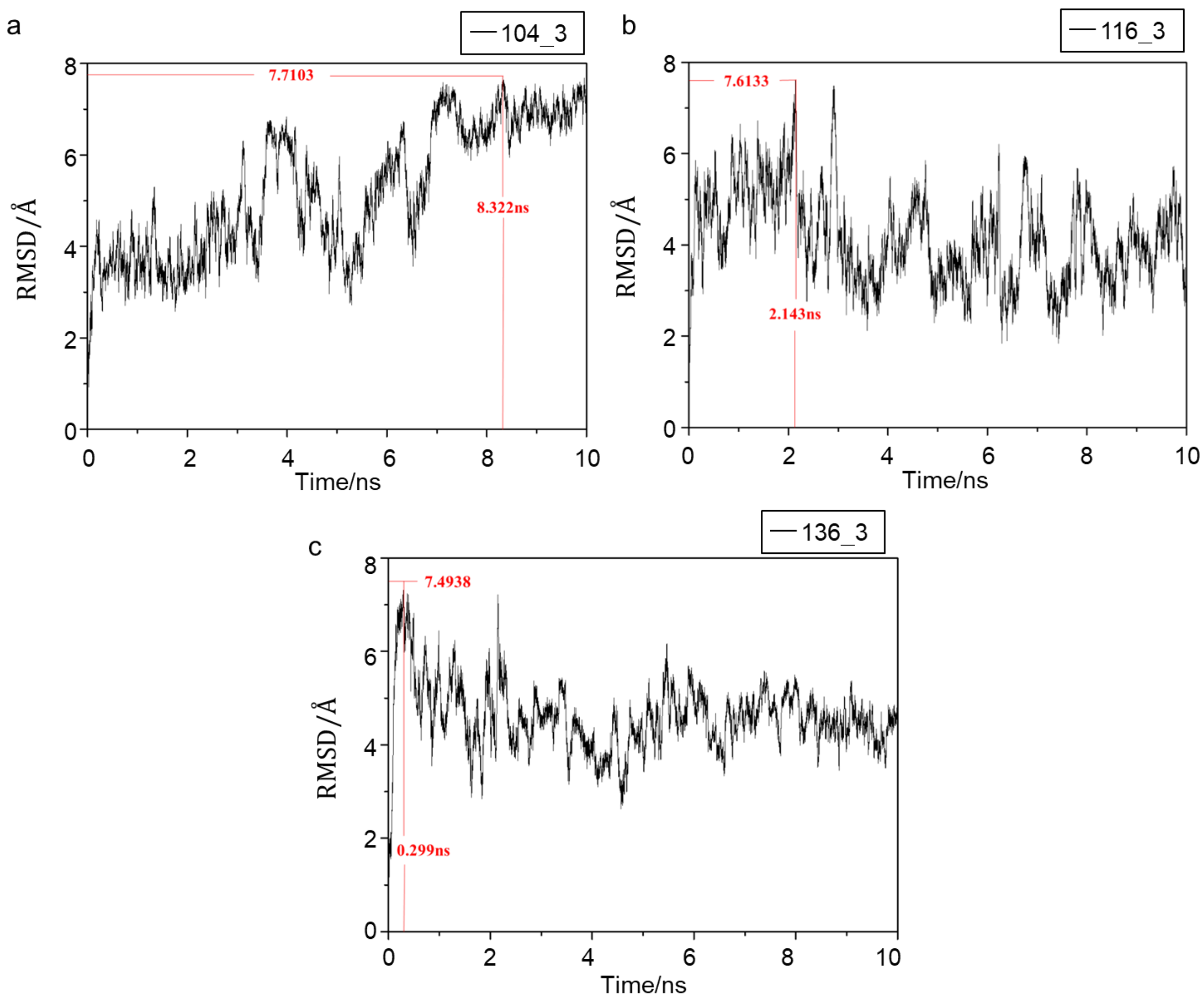

Figure S7. The distributions of the RMSD values for structures: (a) 104_3, (b) 116_3 and (c) 136_3.

References

[1] Frisch, M. J.; Trucks, G. W.; Schlegel, H. B.; Scuseria, G. E.;Robb, M. A.; Cheeseman, J. R.; Scalmani, G.; Barone, V.; Petersson,G. A.; Nakatsuji, H.; Li, X.; Caricato, M.; Marenich, A. V.; Bloino, J.;Janesko, B. G.; Gomperts, R.; Mennucci, B.; Hratchian, H. P.; Ortiz, J.V.; Izmaylov, A. F.; Sonnenberg, J. L.; Williams-Young, D.; Ding, F.;Lipparini, F.; Egidi, F.; Goings, J.; Peng, B.; Petrone, A.; Henderson,T.; Ranasinghe, D.; Zakrzewski, V. G.; Gao, J.; Rega, N.; Zheng, G.;Liang, W.; Hada, M.; Ehara, M.; Toyota, K.; Fukuda, R.; Hasegawa, J.;Ishida, M.; Nakajima, T.; Honda, Y.; Kitao, O.; Nakai, H.; Vreven, T.;Throssell, K.; Montgomery, J. A., Jr.; Peralta, J. E.; Ogliaro, F.;Bearpark, M.; Heyd, J. J.; Brothers, E. N.; Kudin, K. N.; Staroverov, V.

N.; Kobayashi, R.; Normand, J.; Raghavachari, K.; Rendell, A.; Burant,J. C.; Iyengar, S. S.; Tomasi, J.; Cossi, M.; Millam, J. M.; Klene, M.;Adamo, C.; Cammi, R.; Ochterski, J. W.; Martin, R. L.; Morokuma,K.; Farkas, O.; Foresman, J. B.; Fox, D. J. Gaussian 16, revision A.03; Gaussian, Inc.: Wallingford CT, **2016**.

[2] S. Grimme, J. Antony, S. Ehrlich and H. Krieg, *Chem. Phys.* **2010**, *132*, 154104-154123.

[3] Case, D. A.; Ben-Shalom, I. Y.; Brozell, S. R.; Cerutti, D. S.; Cheatham, T. E.; III; Cruzeiro, V. W. D.; Darden, T. A.; Duke, R. E.; Ghoreishi, D.; et al. AMBER **2018**, University of California, San Francisco.

[4] Bayly, C. I., Cieplak, P., Cornell, W. & Kollman, P. A. A well-behaved electrostatic potentialbased method using charge restraints for deriving atomic charges: the RESP model. *J. Phys.Chem*. **1993**, *97*, 10269-10280.

[5] Wang, J. et al. Development and testingof a general AMBER force field. *J. Comput. Chem*. **2004**, *25*, 1157-1174.

[6] a) Y. Hui, X. Yi, F. Hou, D. Wibowo, F. Zhang, D. Zhao, H. Gao, C. X. Zhao, *ACS Nano* **2019**, *13*, 7410-7424; b) A. C. Anselmo, S. Mitragotri, *Adv Drug Deliv Rev* **2017**, *108*, 51-67.

[7] W. J. Liu, X. Y. Zhou, Z. W. Mao, D. H. Yu, B. Wang, C. Y. Gao, *Soft Matter* ***2012***, *8*, 9235-9245.

[8] A. C. Anselmo, M. Zhang, S. Kumar, D. R. Vogus, S. Menegatti, M. E. Helgeson, S. Mitragotri, *ACS Nano* **2015**, *9*, 3169-3177.

[9] M. F. Bedard, A. Munoz-Javier, R. Mueller, P. del Pino, A. Fery, W. J. Parak, A. G. Skirtach, G. B. Sukhorukov, *Soft Matter* ***2009***, *5*, 148-155.

[10] R. Hartmann, M. Weidenbach, M. Neubauer, A. Fery, W. J. Parak, *Angew. Chem. Int. Ed.****2015***, *54*, 1365-1368.

[11] J. Cui, R. De Rose, J. P. Best, A. P. Johnston, S. Alcantara, K. Liang, G. K. Such, S. J. Kent, F. Caruso, *Adv. Mater.* ***2013***, *25*, 3468-3472.

[12] a) M. Yu, L. Xu, F. Tian, Q. Su, N. Zheng, Y. Yang, J. Wang, A. Wang, C. Zhu, S. Guo, X. Zhang, Y. Gan, X. Shi, H. Gao, *Nat. Commun.* **2018**, *9*, 2607; b) P. Guo, D. Liu, K. Subramanyam, B. Wang, J. Yang, J. Huang, D. T. Auguste, M. A. Moses, *Nat. Commun.* **2018**, *9*, 130; c) J. F. Alexander, V. Kozlovskaya, J. Chen, T. Kuncewicz, E. Kharlampieva, B. Godin, *Adv. Healthc. Mater.* **2015**, *4*, 2657-2666.
